# Osteoblasts in Bone Physiology—Mini Review

**DOI:** 10.5041/RMMJ.10080

**Published:** 2012-04-30

**Authors:** Nahum Rosenberg, Orit Rosenberg, Michael Soudry

**Affiliations:** Musculoskeletal Research Laboratory, Division of Orthopedics, Rambam Health Care Campus, and The Rappaport Faculty of Medicine, Technion–Israel Institute of Technology, Haifa, Israel

**Keywords:** Bone, cytokines, hedgehog, mechanotransduction, osteoblast, Wnt

## Abstract

Bone structural integrity and shape are maintained by removal of old matrix by osteoclasts and *in-situ* synthesis of new bone by osteoblasts. These cells comprise the basic multicellular unit (BMU). Bone mass maintenance is determined by the net anabolic activity of the BMU, when the matrix elaboration of the osteoblasts equals or exceeds the bone resorption by the osteoclasts. The normal function of the BMU causes a continuous remodeling process of the bone, with deposition of bony matrix (osteoid) along the vectors of the generated force by gravity and attached muscle activity. The osteoblasts are derived from mesenchymal stem cells (MSCs). Circulating hormones and locally produced cytokines and growth factors modulate the replication and differentiation of osteoclast and osteoblast progenitors. The appropriate number of the osteoblasts in the BMU is determined by the differentiation of the precursor bone-marrow stem cells into mature osteoblasts, their proliferation with subsequent maturation into metabolically active osteocytes, and osteoblast degradation by apoptosis. Thus, the two crucial points to target when planning to control the osteoblast population are the processes of cell proliferation and apoptosis, which are regulated by cellular hedgehog and Wnt pathways that involve humoral and mechanical stimulations. Osteoblasts regulate both bone matrix synthesis and mineralization directly by their own synthetic activities, and bone resorption indirectly by its paracrinic effects on osteoclasts. The overall synthetic and regulatory activities of osteoblasts govern bone tissue integrity and shape.

## INTRODUCTION: BONE

**Figure 1:**
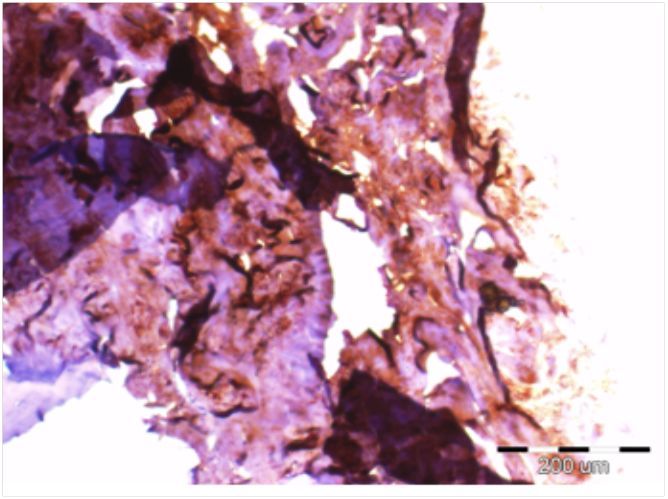
**Microscopic image of immunohistochemical staining for collagen I (brown color) in cancellous bone sample.** Scale bar 200 μm.

**Figure 2:**
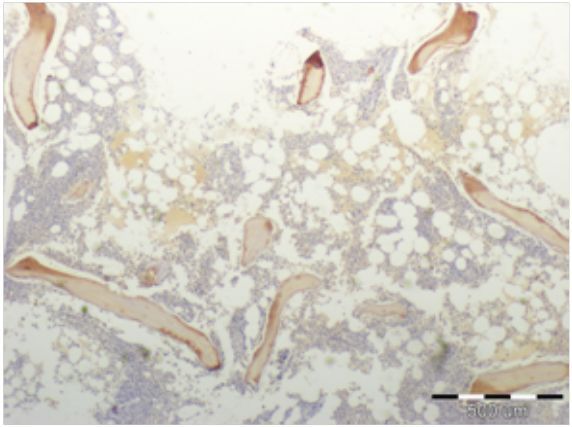
**Microscopic image of immunohistochemical staining for osteocalcin (brown color) in cancellous bone sample.** Scale bar 500 μm.

**Figure 3:**
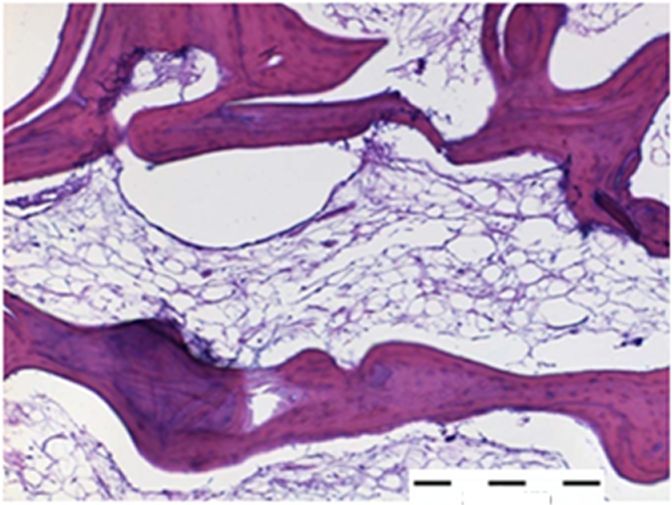
**Microscopic image of normal bone sample (HE staining).** Eosinophilic matrix is surrounded by osteoblasts on its edges. Scale bar 200 μm.

**Figure 4:**
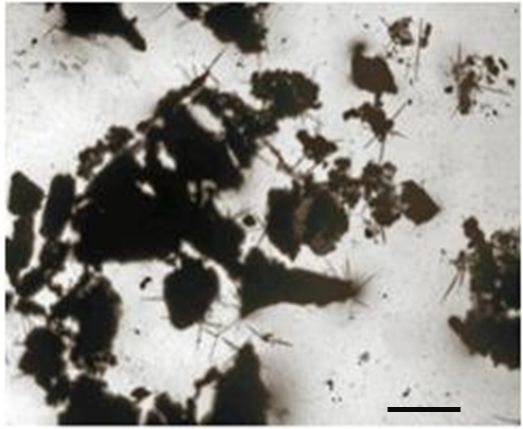
**Microscopic image of Von Kossa staining (calcium bone nodules stained by 5% silver nitrate) adjacent to cultured human osteoblasts.** Scale bar 30 μm.

Bone functions as an anchorage for muscles enabling movement, and as a protective boundary for vital organs such as brain and spinal cord. Its solid characteristics are due to the calcified matrix which is composed of inorganic components of calcium and phosphate, as hydroxyapatite, deposited on organic components, mainly collagen I (Figure 1), and 5% of non-collagenous proteins, such as osteopontin and osteocalcin (Figure 2), etc. The synthesis and calcification of the bone matrix is governed by the osteoblasts (bone-generating cells). The osteoblasts are mostly situated in the matrix boundaries (Figure 3). The matrix mineralization occurs in matrix vesicles along the collagen fibrils (Figure 4). We describe how the osteoblasts regulate mineralization of bone matrix. Since the osteoblasts govern the overall process of bone maintenance, their malfunction can cause bone mass depletion, over-production, or production–resorption imbalance, causing osteoporosis, osteopetrosis, or Paget’s disease of the bone, respectively. Since these pathological conditions are seriously disabling, especially due to their tendency to cause pathological fractures, understanding the cellular regulatory pathways of the osteoblast is crucial for development of therapeutic modalities for treatment of bone diseases.

## BASIC MULTICELLULAR UNIT (BMU)

Bone structural integrity and shape are maintained by removal of old matrix by osteoclasts and *in-situ* synthesis of new bone by osteoblasts.^1^ Resorption and formation are perceived as independent processes but, in reality, they are closely linked within temporary structures called the basic multicellular unit (BMU).^2^ A fully developed BMU consists of a group of osteoclasts, osteoblasts, blood supply, and connective tissue. As the entire BMU moves forward alongside the bone, osteoclasts resorb bone and die by apoptosis. The average life-span of an osteoclast is about 12 days. The resorbed bone is replaced by osteoblast cells synthesizing bone matrix. The life-span of osteoblasts varies from a few to about 100 days.

The osteoblasts are derived from mesenchymal stem cells (MSCs). Circulating hormones and locally produced cytokines and growth factors modulate the replication and differentiation of osteoclast and osteoblast progenitors. The most important locally produced pro-osteoclastic cytokine is a receptor activator of the nuclear factor ligand (RANKL) or NF-kappaB. It is expressed by the MSCs and binds to its receptor (RANK) on osteoclast progenitors to stimulate their differentiation. The maturation of osteoblasts is promoted by growth factors released from the bone matrix during resorption, as well as by growth factors produced by osteoblast progenitors themselves. Many of the growth factors govern the life-span of osteoblasts and osteoclasts by their effects on apoptosis. Bone loss in sex steroid deficiency or following glucocorticoid excess is caused by alteration of bone cell production and shortening of osteoblast life-span, and by osteoclast life-span alterations. Therapies that prevent or reverse osteoporosis act, at least in part, by preventing osteoblast apoptosis and stimulating osteoclast apoptosis. The following is a partial list of hormones that regulate apoptosis in bone cells:

- Estrogen promotes osteoclast apoptosis, but prevents osteoblast apoptosis.^3^,^4^
- Glucocorticoid reduces osteoblast number and has a direct anti-apoptotic effect on the osteoclast.^5^
- Parathyroid hormone (PTH) inhibits osteoblast apoptosis.^6^

## REGULATION OF THE BMU

**Figure 5:**
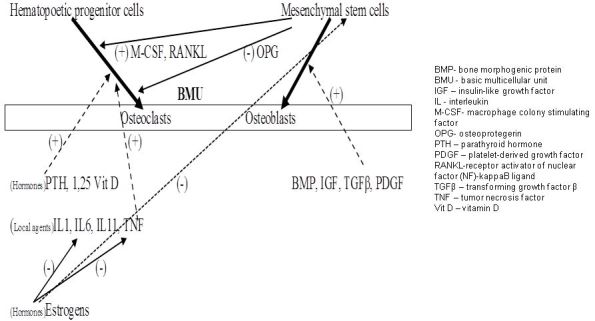
**Interactions between BMU components.**

Bone mass maintenance is determined by the net anabolic activity of the BMU,^7^ when the matrix elaboration of the osteoblasts exceeds the bone resorption by the osteoclasts. The normal function of the BMU causes a continuous remodeling process of the bone with deposition of bony matrix (osteoid) along the vectors of the generated force by gravity and attached muscle activity (Wolff’s law)^8^ and resorption of the bone that is not aligned with these boundaries. A non-physiological propagation of forces along the bones, such as immobilization of a limb by an external device or low gravity condition on one side, or impaired biochemical control of the BMU, as happens in several pathological conditions, will cause an imbalance in the BMU function with subsequent pathological bone resorption (i.e. osteoporosis) or over-production (i.e. osteopetrosis) or both (e.g. Paget’s disease of the bone).^9^ All these conditions can lead to significant disability due to excessive bone fragility, with fractures that fail to heal adequately. The genesis of the osteoclast–osteoblast unit from the progenitor stem cells is regulated by local and hormonal factors with mutual feedback control (Figure 5).

The macrophage colony-stimulating factor (M-CSF), receptor activator of nuclear factor (NF)-kappaB ligand (RANKL), and osteoprotegerin (OPG) are local factors that are secreted by the mesenchymal progenitors of the osteoblasts. The former two agents positively regulate the osteoclasts’ transformation from their progenitors. The OPG has a negative feedback action on osteoclast formation by the RANKL inactivation. The osteoclastogenetic local and hormonal agents act in parallel by inducing the osteoclast formation directly and by inactivating the OPG.

The programmed degradation of the BMU components, apoptosis, is also controlled by the progenitor cell products. Osteoclast apoptosis is negatively controlled by the RANKL and induced by the OPG.^10^ The estrogens and PTH have a protective anti-apoptotic role on the osteoblasts and their precursors.^11^

From these complex interactions it is clear that, although the components of the BMU originate from the two distinct groups of the progenitor cells, the cells from the mesenchymal origin (MSCs) govern the whole BMU function by their positive and negative feedback signals. In order to control these cellular processes, a thorough understanding of the metabolism of the cells of mesenchymal origin, i.e. osteoblasts, is crucial. The appropriate number of the osteoblasts in the BMU is determined by:

1. The differentiation of the precursor stem cells into mature osteoblasts
2. Their proliferation with subsequent maturation into metabolically active osteocytes
3. Osteoblast degradation by apoptosis

Thus, the two crucial points to target when planning to control the osteoblast population are the processes of cell proliferation and apoptosis.

## REGULATION OF OSTEOBLAST DEGRADATION BY APOPTOSIS

In general, apoptosis in mammalian cells is controlled by two signaling pathways. One is initiated by plasma membrane tumor necrosis factor (TNF) receptors and the other through mitochondrial membrane depolarization with subsequent release of cytochrome C. Both pathways activate the cascade of proteolytic enzymes of the caspase type with subsequent cellular autolysis.^12^ Most of the growth factors and anabolic hormones, such as fibroblast growth factor (FGF), insulin-like growth factor (IGF), interleukin (IL)-6, PTH, sex steroids, and calcitonin, have protective anti-apoptotic effects in the osteoblasts.^13^–^15^ There are three main factors that are known to be apoptosis-inductive in osteoblasts:

- TNF, through activation of plasma membrane receptors
- Glucocorticosteroids
- Bone morphogenic protein 2 (BMP2), by cytochrome C release from the mitochondria^16^

**Figure 6:**
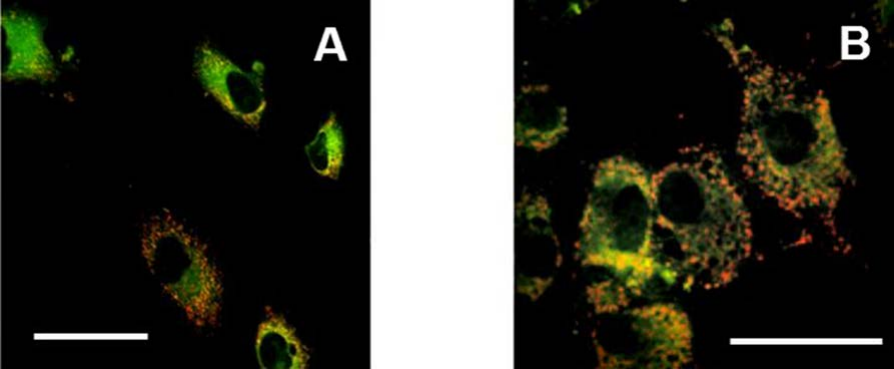
**Examples of microscopic images of cells stained by JC-1.** **A:** Control cultured osteoblasts. **B:** Cultured osteoblasts treated by pro-apoptotic agent (FGIN-1-27). Greater red color staining is apparent in cells treated by pro-apoptotic agent **(B)** in comparison to untreated osteoblasts **(A)**, indicating a decrease in the mitochondrial membrane potential. Confocal microscopy. Scale bar 30 μm.

**Figure 7:**
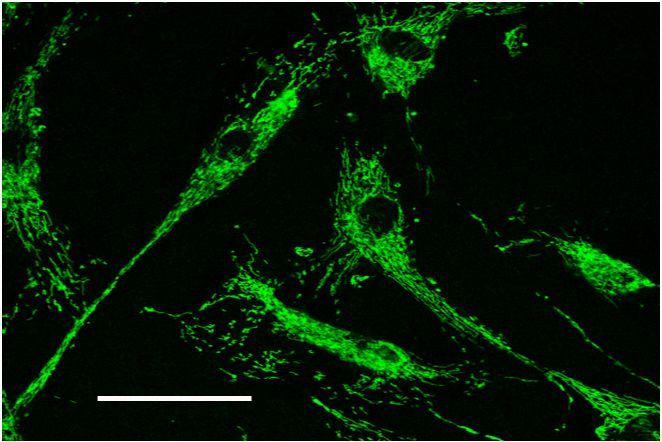
**Microscopic imaging (confocal microscopy) of human osteoblasts stained by Mito Tracker Green stain, which is specific to mitochondria.** The high content of mitochondria in human osteoblasts is evident. Scale bar 10 μm.

In the mitochondrial apoptotic pathway, the basic process involves depolarization of the inner mitochondrial membrane with subsequent increase of permeability and leakage of the outer membrane. This process involves an increase of permeability of the voltage-dependent anion channel (VDAC) on the mitochondrial outer membrane with parallel adenine nucleotide translocator (ANT) disruption on the inner membrane.^17^ This process involves interactions of proteins of mitochondrial permeability transition pores (MPTP),^18^ which recently were found to be very abundant in osteoblasts.^19^ A pro-apoptotic agent causes the collapse of the mitochondrial membrane potential (ΔΨ m) (Figure 6). Since the osteoblasts are highly metabolically active cells, they are rich in mitochondrial content (Figure 7) and, therefore, potentially susceptible to mitochondrial apoptotic threats, but it is not clear what apoptotic pathway is predominant in pathological conditions such as osteoporosis of its different types.^20^

## REGULATION OF OSTEOBLAST MATURATION AND PROLIFERATION

The conversion of mesenchymal stem cells into osteoblasts and the later maturation and proliferation are regulated by the hedgehog and Wnt signaling pathways.^21^,^22^ The Wnt family of proteins interacts with cellular surface receptors, frizzled and Lrp 5/6, inducing the canonic cytoplasmatic signaling pathway. Alternatively, the Wnt pathway can be activated by mechanical deformation of the osteoblast by external forces, via activation of cytoskeletal components, i.e. non-canonical pathways that involve calcium flux into cells.^23^ Therefore, the dual ability of osteoblasts to activate the Wnt signaling, either humoral or mechanical, explains the sensitivity of these cells to mechanical stimuli and to biochemical agents (growth factors and cytokines). The extent of this dual regulation effect is unique to osteoblasts.

The hedgehog (Hh) signaling pathway functions upstream to the Wnt cellular effects, and its main role is in induction of initial maturation of MSCs toward an osteoblastic lineage.^22^ There are several Hh ligands that are involved in osteoblast maturation; the most investigated are Sonic hedgehog (Sh) and Indian hedgehog (Ih). These ligands release the inhibitory effect of the cell membrane protein Ptch1 on the Smo membrane protein. When uninhibited the latter induces the activation intracellular signaling pathway for activation of several genes (transcription factors) that are involved in cellular maturation, e.g. gli1, hip1, and others.^22^ Therefore the Hh and Wnt ligands cause synergistic effect on MSCs’ maturation into osteoblasts.

## CONCLUSION

Osteoblasts regulate directly the bone matrix synthesis and mineralization by their synthetic activities, and indirectly they regulate the bone resorption by paracrinic effects on osteoclasts. The overall synthetic and regulatory activities of osteoblasts govern bone tissue integrity and shape. Thus, in the development of treatment modalities for several serious pathologic conditions, e.g. osteoporosis, osteosarcoma, etc., the ability to intervene in the osteoblast metabolism, maturation, and proliferation is crucial. The above-presented humoral, mechanical, and cellular signaling pathways that regulate these activities in osteoblasts are the natural targets for the treatment intervention in pathological conditions.

## Abbreviations:

ANT: adenine nucleotide translocator;
BMP2: bone morphogenic protein 2;
BMU: basic multicellular unit;
FGF: fibroblast growth factor;
Hh: hedgehog;
Ih: Indian hedgehog;
M-CSF: macrophage colony-stimulating factor;
MPTP: mitochondrial permeability transition pore;
MSC: mesenchymal stem cell;
OPG: osteoprotegerin;
PTH: parathyroid hormone;
RANK: receptor activator;
RANKL: receptor activator of nuclear factor (NF)-kappaB ligand;
Sh: Sonic hedgehog;
TNF: tumor necrosis factor;
VDAC: voltage-dependent anion channel.
